# Marigold suspension cell culture as production system for recombinant DsRed and brazzein

**DOI:** 10.1007/s00299-026-03982-8

**Published:** 2026-09-30

**Authors:** Matthias Buntru, Alexander Croon, Jürgen Müller, Marika Hanke, Helga Schinkel

**Affiliations:** Fraunhofer Institute for Molecular Biology and Applied Ecology IME, Forckenbeckstrasse 6, 52074 Aachen, Germany

**Keywords:** Recombinant protein production, Suspension culture, Brazzein, LC–MS/MS, DsRed, Proteases

## Abstract

**Key Message:**

Newly established Calendula officinalis suspension culture with low protease activity in the supernatant is transformed and produces high yields of recombinant proteins both in the cytosol and in the apoplast.

**Abstract:**

A marigold (*Calendula officinalis*) suspension cell culture (CR1) was established from very young root tissues. Protease activity was checked in the cell culture supernatant (SN) and was found to be very low. To determine if CR1 could be a suitable production platform for recombinant proteins, a transformation was performed. Using *Agrobacterium tumefaciens*, CR1 was transformed with a construct containing two cassettes: one coding for a red fluorescent protein (DsRed) targeted to the endoplasmic reticulum and the other coding for the sweet protein brazzein, targeted to the apoplast. Suspension cultures were initiated from calli that exhibited strong DsRed fluorescence. A brazzein standard was prepared from the SN of one transformed CR1 line by purifying brazzein and subsequent quantification via tryptic digest and mass spectrometric comparison with a commercial brazzein-derived peptide of known concentration. This standard was used to determine the brazzein concentration in seven different transgenic CR1 transformation events by liquid chromatography–tandem mass spectrometry (LC–MS/MS), with the highest concentration measured at 24.5 µg/ml. Quantification of DsRed production in the cells of the same cultures showed up to 1322 µg/g fresh weight of DsRed (equivalent to 34.9% of total soluble protein). CR1 is potentially an excellent novel alternative to BY2 cells for the production of recombinant proteins.

**Supplementary Information:**

The online version contains supplementary material available at https://doi.org/10.1007/s00299-026-03982-8.

## Introduction

Plant cell culture has been a field of study for some time (Haberlandt 1902), although it gained wider recognition within the scientific community first in 1962 with the publication introducing the Murashige and Skoog medium (MS medium) (Murashige & Skoog 1962). Plant cell culture was subsequently used for various purposes, including plant breeding (McCoy & Bingham 1977), micropropagation of plants (Kitto & Janick 1985), and secondary metabolite production (Zenk 1991). The establishment of tobacco Bright Yellow 2 (BY2) cells (Kato et al. 1972a, 1972b) marked a significant advancement in plant cell culturing. BY2 cells, which are rapidly growing and easily manipulated, allow for genetic engineering, gene expression studies, and the production of valuable compounds such as secondary metabolites or recombinant proteins. For large-scale production of recombinant proteins, prokaryotic systems are usually more suitable due to easy setup, high protein production rates, and cost efficiency. However, some proteins need to be produced in a eukaryotic host to enable post-translational modifications (e.g., glycosylation) or complex folding. For the production of such proteins, BY2 cells have been used (Schiermeyer et al. 2005; Sack et al. 2007), as well as other plant suspension cells (Santos et al. 2016). A notable example is taliglucerase alfa, sold under the brand name Elelyso; this therapeutic protein for the treatment of Gaucher disease is produced in carrot suspension cells (Shaaltiel et al. 2007). Moreover, the production of secondary metabolites in plant cell cultures is gaining traction (Wawrosch & Zotchev 2021). This sustainable method avoids overharvesting wild plants and is also less affected by environmental and geopolitical disturbances. In contrast to whole plants, a cell culture in a bioreactor can be easily manipulated by physical factors (temperature, stirring etc.) and chemical substances (medium, feeding of intermediates) (Murthy et al. 2024). One advantage of using plant cells to produce secondary metabolites is the multitude of substances that are naturally produced in plants (Bills & Gloer 2016), meaning there is a multitude of pathways readily available. This makes plant suspension cells a valuable tool for the production of intricate molecules needed to address the challenges of the twenty-first century.

While BY2 cells are still a widely used cell line for research and recombinant protein production, a new plant suspension culture that shares all the positive aspects—such as robustness, quick growth, and ease of genetic manipulation—but superior to BY2 cells in regard to age (i.e., cell cycles) and potentially the presence of secondary metabolism pathways was the aim of this study. Additionally, a tobacco-derived cell line may face challenges regarding customer acceptance when used for the production of food-related substances (Krasteva et al. 2020). Marigold (*Calendula officinalis*), a well-known therapeutic herb with a positive connotation and a great number of secondary metabolites (Shahane et al. 2023), was chosen for the establishment of a new plant suspension culture. The resulting culture was then assessed for its suitability as a production system for recombinant proteins.

## Experimental procedures

### Plants, bacteria, vector used

Seeds of *Calendula officinalis* L. (CO030) were obtained from Jelitto (Schwarmstedt, Germany). The *Agrobacterium tumefaciens* strain GV3101::pMP90RK(GmR, KmR, RifR) (Koncz & Schell 1986), carrying the vector pTRAkc_LPH-Braz_rfp-ERH, was used for the transformation of the calendula suspension cell line. The vector contains the gene coding for the tetrameric red fluorescent protein DsRed from *Discosoma* spec. (GenBank AF168419), and in a second cassette, the gene coding for the sweet protein brazzein from *Pentadiplandra brazzeana* (GenBank EU883595), both under the control of the 35S promoter. For a detailed description, see Fig. S1.

### Generation of suspension cultures

The seeds were sterilized by first washing them for 2–3 min in 70% ethanol and then for 5 min in 1.4% (w/v) sodium hypochlorite. Afterward, they were thoroughly washed with sterile water and placed on agar plates containing 2.15 g/L MS medium (M0221, Duchefa, Haarlem, the Netherlands) and 0.8% (w/v) plant agar (Phytoagar P1003, Duchefa). The plates were sealed and placed at 21 °C with a 16 h light/8 h dark cycle. When seed germination was visible, the emerging rootlets were cut into small pieces using a scalpel, and these pieces were then placed on MS1 medium: 4.3 g/L MS medium (M0221, Duchefa), 3% sucrose, 0.2 g/L KH_2_PO_4_, 1 mg/L thiamine, 0.1 g/L myo-inositol and 0.2 mg/L 2,4D, pH5.8, containing 0.8% plant agar. The plates with the plant material were then incubated at 21 °C in the dark. When callus became visible, it was transferred to fresh MS1 plates and incubated as described earlier. Suspension cultures were initiated as soon as there was enough white, soft callus for a small culture. The callus was placed on MS1 medium and incubated at 26 °C, 140 rpm, in the dark with weekly subcultures. Two months later, a twice-weekly passaged culture was established using cells from the weekly passaged culture. The new culture was named Calendula Root 1 (CR1).

### Protease activity in cell culture medium

A weekly passaged CR1 culture, harvested on day 7 with a packed cell volume (PCV) of 50%, was used to assess proteolytic activity in the medium. For comparative purposes, a BY2 cell culture (PCV 55%) cultivated in the same medium under the same conditions was analyzed in parallel. The supernatants (SNs) were harvested by vacuum filtration. Protease activity was analyzed as described previously (Schiermeyer et al. 2005) with modifications. Gelatin, used as a substrate for proteolysis, was copolymerized with a 10% SDS–polyacrylamide gel at a final concentration of 0.1% (w/v). Gels were run at 100 mV for 2 h, and after electrophoresis, they were washed twice with washing buffer (1% (v/v) Triton X-100 in incubation buffer) for 20 min at room temperature. The gels were then incubated overnight in incubation buffer (50 mM Tris–HCl, 5 mM CaCl_2_, 100 µM ZnCl_2_, pH 7.6) at room temperature with gentle agitation. Subsequently, the gels were washed briefly with water and stained with Coomassie Brilliant Blue (CBB) according to Wong et al. (2000), revealing areas of proteolytic gelatin degradation during the destaining process.

### Transformation of CR1

For the transformation of CR1, the twice-weekly passaged culture was used, harvested on day two with a PCV of 39%. The cells were handled as previously described (Cocultivation on solid ABM-MS medium; Buntru et al. 2025) with minor modifications. Specifically, the cocultivation was performed in the dark. After 2 days, the cells were washed twice with MS1 supplemented with 250 µg/mL ticarcillin disodium/clavulanate potassium and then maintained in the same medium in six-well plates, shaking at 120 rpm for 3 days at 25 °C with 70% humidity for regeneration. The cell suspension was then plated on MS1a plates (MS1 containing 0.8% plant agar, 30 mg/L kanamycin, and 250 mg⁄L ticarcillin disodium/clavulanate potassium). Plates were incubated at 22 °C in the dark.

When calli emerged, they were checked for DsRed fluorescence using a cold light source with 1 m fiber optics and a green excitation filter (Schott, Mainz, Germany) along with a red emission filter (light red 182; LEE filters, Andover, UK). Fluorescent calli were picked and transferred to 24-well plates containing MS1a. The calli were then passaged every 3–4 weeks on fresh MS1a, with the kanamycin concentration in the medium gradually increased to a final concentration of 100 mg/L.

Calli that produced a high amount of DsRed appeared pink to the naked eye (Rademacher et al. 2019). Pink calli were used to initiate suspension cultures by placing approximately 200 mg of fresh callus into 3 mL of MS1 medium containing 100 mg/L kanamycin and 250 mg⁄L ticarcillin disodium/clavulanate potassium in a small Erlenmeyer flask. The flask was then incubated at 26 °C, 140 rpm, in the dark, with regular subculturing and a concurrent increase of the culture volume to 50 mL. At harvest, the cultures were separated into cells and SNs by applying vacuum, where the cells were collected on filter paper in a Büchner funnel while the SN was recovered from the suction flask. Both fractions were then aliquoted and frozen at − 20 °C.

### Visualization of brazzein

Suspension culture SNs from CR1 transformation events 16, 18, 74, 80, 86, 141, 155, and CR1 Wt were analyzed on sodium dodecyl sulfate polyacrylamide (SDS-PAA) gels. The frozen SNs were thawed, and 200 µL were dried by vacuum centrifugation, redissolved in 40 µL (8 M urea in 50 mM ammonium bicarbonate) and alkylated (see text S1).

SDS-PAA gels were prepared according to Schägger (2006). The gels consisted of a separating gel (17% polyacrylamide) and a stacking gel (4% polyacrylamide), with the first prepared using Rotiphorese Gel 40 (29:1) and the latter prepared with Rotiphorese Gel 30 (37.5:1) (both Carl Roth, Karlsruhe, Germany). Neither glycerol nor urea was used.

Samples were mixed with NuPAGE LDS sample buffer and NuPAGE sample reducing agent (both Thermo Fisher Scientific, Darmstadt, Germany) according to the manufacturer’s instructions, incubated at 95 °C for 5 min, cooled down, and loaded onto the prepared Schägger gel. Two different size markers were used: spectra multicolor low-range protein ladder (Thermo Fisher Scientific) and a homemade mix of aprotinin (Carl Roth), ribonuclease A (GE Healthcare, Freiburg, Germany), and lysozyme (Sigma, Taufkirchen, Germany). The gel was run at a constant voltage of 30 V for 15 min, then 80 V for 48 min, followed by 130 V for 156 min, and finally 200 V for 8 min. Proteins on SDS-PAGE were stained with CBB according to Wong et al. (2000).

### Quantification of brazzein in cell culture supernatants by top-down LC–MS/MS

A top-down liquid chromatography–tandem mass spectrometry (LC–MS/MS) approach was used to analyze the amount of brazzein in the SNs of seven different transformation events of CR1. SNs (100 µL each) were precipitated with acetone, reconstituted, desalted and concentrated to a final volume of 50 µL (see Text S1).

Calibration was performed by spiking a newly prepared brazzein standard (for preparation and characterization of standard see supplemental text S1) into CR1 Wt SN at various concentrations, ranging from 1 to 50 µg/mL. The LC was conducted as described (Text S1) for the integrity analysis of the brazzein standard. Brazzein was monitored in targeted selected ion monitoring mode at a resolution of 120,000 at *m/z* 200. The isolation window was *m/z* 1.5, the NCE was set to 30, and the automatic gain control (AGC) target was 5×10^4^. Brazzein detection was achieved by measuring the sevenfold positively charged ion at *m/z* 925.6880. Data processing and peak integration were performed using Freestyle Software (ver. 1.8.65.0; Thermo Fisher Scientific). The samples were quantified by performing a linear regression analysis.

### DsRed extraction and quantification

Established suspension cultures of stably transformed CR1 cells and CR1 Wt cells were grown in a volume of 50 mL for 7–15 days (Table 1). At harvest, SNs and cells were separated by vacuum filtration as described above. The total amount of SN and cells from each culture was measured and aliquoted (cells approximately 200 mg; SN 200 µL) and frozen at − 20 °C. All SNs and cells were analyzed for DsRed concentration in one experiment. The frozen suspension cells (4 aliquots for each culture) were mixed with 2.5 times the amount (v/w) of phosphate-buffered saline (PBS) in 2 mL Eppendorf tubes and thawed at room temperature. Samples were sonicated using the Q-Sonica, model Q700 (Sonica LLC, Newton, CT, U.S.A.), equipped with a microtip at 40% amplitude, with a pulse on/off of 5 s, and a process time of 20 s. While sonication was performed, the tubes were placed in an ice-cold metal block. Samples were then centrifuged at 16,100 × g and 4 °C for 30 min, and the supernatant was transferred to a new tube. Quantification was done by diluting the supernatant 1:10 with PBS and quantifying the fluorescent signal from DsRed using a Synergy H1 microplate reader (BioTek, Winooski, USA) and an in-house produced DsRed standard (Lobedann 2009). The obtained values were normalized to the biomass of the respective suspension cells. Culture supernatants were also diluted 1:10 and analyzed in the same way. Total soluble protein (TSP) was measured in cell extracts and SNs of the Wt and transformation events 16, 74, and 155 using the Pierce Coomassie Protein Assay Kit (23,200, Thermo Fisher Scientific), with bovine serum albumin used as reference.

**Table 1:** Production of DsRed and brazzein in selected transformation events

(a) Suspension culture of transformation event	Culture duration [days]	Cells [g]	SNs [mL]	DsRed		Brazzein [µg/mL SN]
				Cells [µg/g] FW	SNs [µg/mL]	
16	7	13.7	33	176 ± 25	0.6	4.1
18	12	9.8	35	202 ± 32	11.3	6.6
74	15	3.6	38	541 ± 112	0	24.5
80	14	5.9	39	481 ± 43	2.2	12.0
86	13	7.1	30	461 ± 18	0.7	8.3
141	7	6.6	36	550 ± 75	2.8	9.6
155	14	3.6	40	1322 ± 163	6.7	11.2
Wt	7	7	37	n.d	n.d	n.d

(b)		
Transformation event	Brazzein [µg/ml/day]	DsRed [µg/g fw/day]
16	0.59	25.1
18	0.55	16.8
74	1.63	36.1
80	0.86	34.4
86	0.64	35.5
141	1.37	78.6
155	0.8	94.4

Seven suspension cultures of transformation events and the Wt suspension culture were analyzed for cell fresh weight, culture supernatant volume, amount of DsRed and brazzein. Cultivation time varied so that at least 3.5 g cell fresh weight could be harvested. All cultures had a starting volume of 50 mL. (a) Measurements done at harvest, (b) normalized production metrics for brazzein/DsRed in the analyzed transformation events. transf. event: transformation event; n.d.: not detected

## Results

### Generation of suspension cell line

**Fig. 1:**
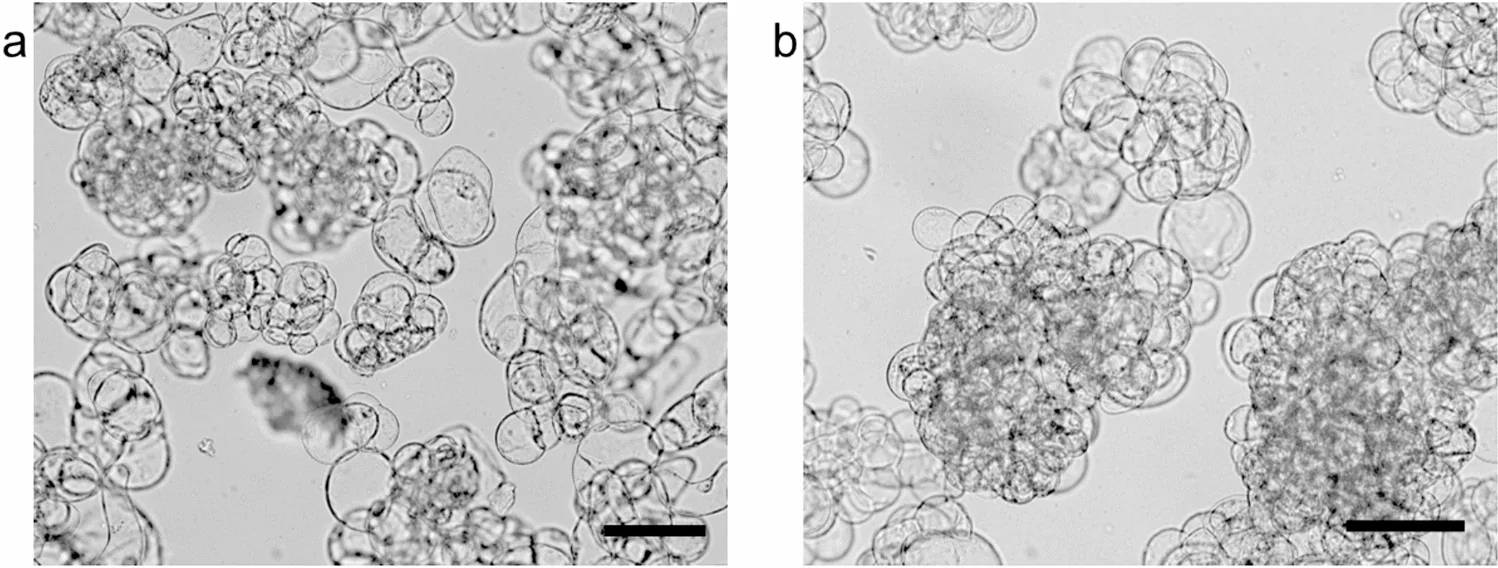
Microscopic pictures of CR1.** a** CR1, twice-weekly passaged, day 3, PCV 53%; **b** CR1, weekly passaged, day 7, PCV 50%. Scale bar: 300 µm

Seeds germinated after 6 days, and rootlets were cut into small pieces and placed on callus induction plates. After a few days, a soft white callus began to grow on some of the root pieces. The callus was passaged twice to fresh plates, and a suspension culture in MS1 medium was started 35 days after the first observation of callus formation. Both the callus and suspension culture are whitish in color; however, with age, both turn brownish-black (Figure S2). The culture CR1 was maintained in parallel under two different passaging regimes, being passaged once a week and twice weekly. When passaged weekly, inoculation with 2% cells led to a PCV of 50–60%, very similar to BY2 cells (see also cultures used for protease activity). CR1 cultures grew homogeneously and the cell size was estimated to be 50–300 µm, with mostly round cells (Fig. 1).

### Protease activity in SN of CR1

**Fig. 2:**
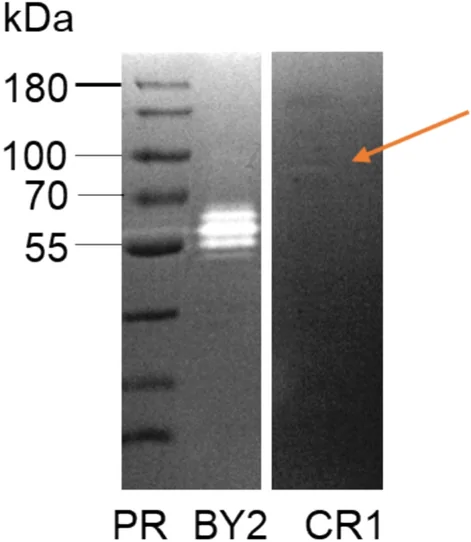
In-gel protease assay of cell culture supernatants. CR1 culture medium and BY-2 culture medium, both from 7-day-old Wt cultures, were separated on a 10% SDS–polyacrylamide gel containing 0.1% (w/v) gelatin. After electrophoresis, the gel was washed in 2.5% (v/v) Triton X-100 and then incubated for 17 h at room temperature in protease buffer. Staining with CBB followed. Clear bands indicate where the gelatin has been proteolytically degraded, resulting in no staining with CBB, with an orange arrow pointing out the weak band in CR1. PR: PageRuler prestained protein ladder (Thermo Fisher Scientific); BY-2: BY-2 suspension cell supernatant; CR1: CR1 suspension cell supernatant

CR1 SN was analyzed for protease activity on a zymogram with gelatin as the substrate. Only minimal proteolytic degradation was observed at a position of approximately 80 kDa. In the same zymogram, BY2 cell culture SN was analyzed, revealing five distinct degradation bands at 50–68 kDa and a weak degradation signal at 100 kDa (Fig. 2).

### Transformation of suspension cells CR1

CR1 suspension cells were successfully transformed 8 months after the suspension culture was initiated. Ten days after the transformed cells were plated on selection medium, the first 72 fluorescent calli were picked and transferred to 24-well plates with fresh selection medium. Five days later, another 84 fluorescent calli were picked and treated similarly. The calli exhibited variation in DsRed production as well as in growth; some calli grew much faster than others (Fig. S3a). To give some idea of the stability of the transgene expression, pictures of three calli, taken 38 passages (roughly 2 years) later, are shown as well (Fig. S3b). Furthermore, some calli were browning (similar to the Wt calli), which was detrimental to the cells.

Establishment of suspension cultures was easily achieved by placing approximately 200 mg of fresh callus in selection medium. After 2 weeks, the suspension cultures were growing homogeneously; however, as with the calli, growth speed varied considerably, as did culture density. While some transformation events (e.g., 141; see Table 1) grew to the same density as the Wt in the same time frame, most cultures of transformed events grew slower and never reached the same cell density as the Wt culture.

### Integrity analysis and quantification of the brazzein standard

**Fig. 3:**
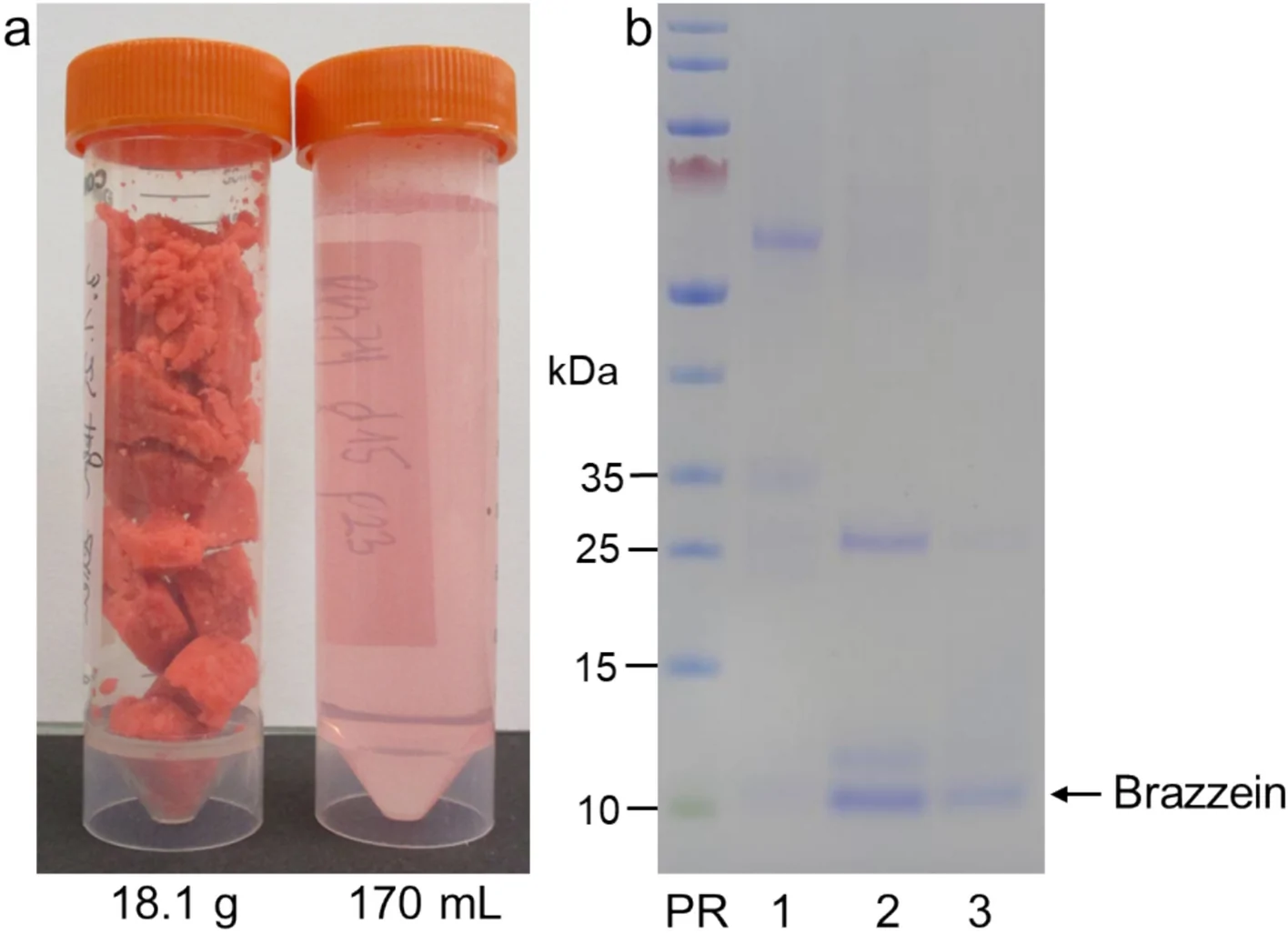
Preparation of a brazzein standard from the cell culture supernatant of transformation event 74**.** The supernatant (SN) of culture 74 was dialyzed against citrate buffer (5 mM, pH 4) and subjected to cation exchange. The fractions with the most brazzein were combined, dialyzed against PBS, and then filtered using a centrifugal filtration device (MWCO 10 kDa) with brazzein ending up in the filtrate. **a** Culture 74 (200 mL), harvested after 15 days; under the picture, the harvested amounts of SN (left) and cells (right) are noted. The cells produced large amounts of DsRed. Seventy mL of the SN were used for the preparation of the standard. **b** Coomassie-stained SDS-PAGE showing the purification of the brazzein standard. 1: Dialyzed culture 74 SN; 2: combined brazzein elution fraction after cation exchange; 3: filtrate after use of the centrifugal filtration device (MWCO 10 kDa). PR: PageRuler prestained protein marker

The brazzein standard was prepared from 70 mL of SN 74 (Fig. 3a). After cation exchange chromatography and ultrafiltration, a clear enrichment of brazzein was observed as a result (Fig. 3b), although other proteins were still present in the standard.

**Fig. 4:**
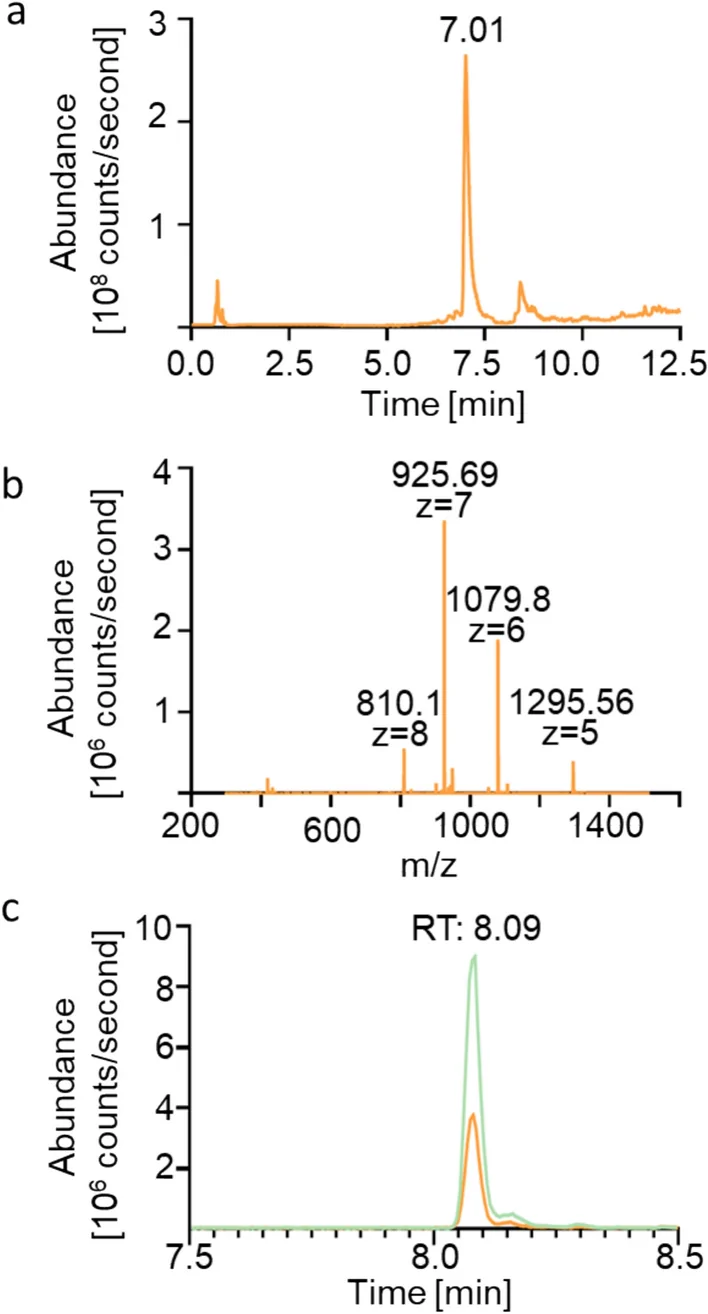
Integrity and quantification of the prepared brazzein standard. **a** TIC chromatogram from 0 to 12.5 min of data-dependent acquisition from *m/z* 300 to 1500 of top-down analysis, showing the purity of the brazzein-enriched in-house reference standard; brazzein elutes at 7.01 min. **b** Mass spectrum from 6.88 to 7.21 min of top-down analysis of brazzein showing the most abundant species of brazzein at [M+7H]^7+^. **c** Overlay of TIC signal *m/z* 781.31 (green signal; RT 8.09 min, peptide from tryptic digest of produced brazzein) and *m/z* 791.31 (orange signal; RT 8.09 min, isotopically labeled peptide), which were used for the bottom-up quantification of brazzein.

Analyzing the prepared standard via LC–MS/MS revealed one major peak at 7.01 min (Fig. 4a), with the main signal at *m/z* of 925.69 for the sevenfold-charged brazzein, and minor signals at *m/z* 1079.8 (sixfold-charged), *m/z* 810.1 (eightfold-charged), and *m/z* 1295.56 (fivefold-charged) (Fig. 4b), all of which were deconvoluted to a molecular weight of 6473 Da, which is the correct average mass of brazzein. The quantification of the brazzein amount in the standard was performed via a trypsin digest and comparison to a commercial peptide of a defined concentration (Fig. 4c); the brazzein standard contained 0.21 ± 0.02 mg/mL brazzein. The volume of the final brazzein standard in PBS was 4.3 mL. The standard was aliquoted and stored at − 80 °C until further use.

### Brazzein visualization and quantification

**Fig. 5:**
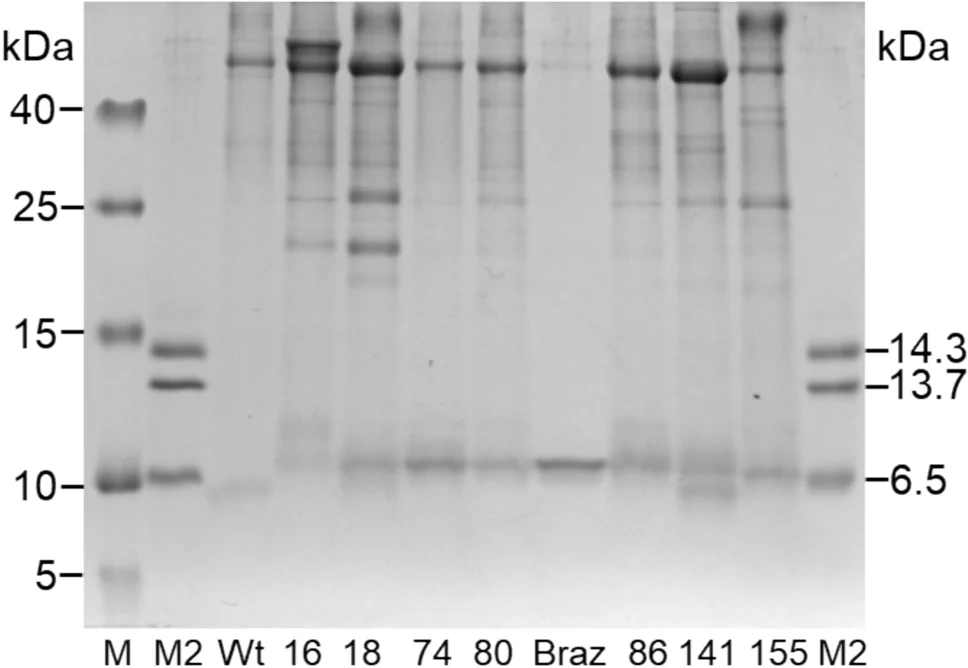
Visualization of brazzein in supernatants of transformed CR1 cells. Suspension cultures of seven CR1 transformation events and the Wt were harvested and separated into cells and supernatants by vacuum filtration. Aliquots of the supernatant were 5× concentrated via vacuum centrifugation and used to visualize brazzein on a 17% Schägger gel, stained with CBB. Numbers under the gel represent different transformation events. Brazzein is detected at 6.5 kDa. M: Spectra™ multicolor low-range protein ladder (Thermo Fisher Scientific); M2: homemade marker consisting of aprotinin (6.5 kDa), ribonuclease A (13.7 kDa), and lysozyme (14.3 kDa); Braz: brazzein standard

The construct used for transformation targeted brazzein to the apoplast. Therefore, the SNs of seven transformation events and the Wt were collected by vacuum filtration. For a quick detection of brazzein, the SNs were concentrated (5x) and then subjected to SDS-PAGE with subsequent CBB staining (Fig. 5). A band at the expected size of brazzein (6.5 kDa) was detected in all analyzed transformation events but not in the Wt. We used the protein aprotinin as an additional size standard after observing that the commercial protein size marker was incorrect at this small size.

The quantification of brazzein in the SNs of the selected transformation events was performed by top-down LC–MS/MS analysis. Following concentration by acetone precipitation and a desalting step of the SNs to reduce matrix effects, brazzein was ionized and detected as a sevenfold-charged ion. To address potential residual matrix effects, we carried out external calibration by spiking our in-house standard into Wt SN and subjected it to the same sample preparation as applied to the samples (Fig. 4S). The concentration of brazzein in the analyzed SNs ranged from 4.1 to 24.5 µg/mL (Table 1), with an average of 10.5 µg/mL.

### DsRed quantification

The construct used for transformation targeted DsRed to the endoplasmic reticulum (ER). Therefore, suspension cells were extracted for the quantification of DsRed. The cells analyzed belonged to the same cultures as the SNs used for brazzein quantification. The amount of DsRed in the cells varied between 176 µg/g fresh weight (fw) (transformation event 16) and 1322 µg/g fw (transformation event 155), with an average of 533 µg/g fw (Table 1a). For improved interpretability, Table 1b shows normalized productivity metrics for both brazzein and DsRed. We also determined TSP in the cell extracts of Wt cells and transformation events 16, 74, and 155. TSP was very similar in all the cell extracts, ranging from 0.9 to 2.0 mg/mL. The DsRed production calculated as a percentage of TSP was 7.5% for event 16, 11.5% for event 74, and as much as 34.9% for event 155, clearly exceeding production rates for other recombinant proteins described in the literature (Santos et al. 2016). To illustrate the high production of DsRed, one extract from each transformation event (of four prepared and measured) is shown in Fig. S5.

DsRed was also measured in the SNs of the same cultures, where values of 0–11.3 µg/mL DsRed were recorded. Relative to the amount of DsRed detected in the cells, between 0 and 19.9% of the DsRed was detected in the SN, with only two lines (18 and 155) having more than 3% of the DsRed leaked from the cells to the SN.

To get an idea of the consistency of TSP and recombinant proteins produced in the wt and the three transformation events 16, 74 and 155, we calculated the total yield for each of the four mentioned 50 ml cultures (Table S1).

## Discussion

A novel suspension cell culture, CR1, was successfully established by generating callus on rootlets of newly germinated marigold seeds and using this fresh callus material to start a suspension culture. CR1 grew quickly in a homogeneous manner and was passaged once weekly to maintain the culture. When the culture was kept for a longer period, it became brownish-black, similar to BY2 cultures (Islam et al. 2023), where the color change was due to the formation of phytomelanin, a pigment well-known to be produced by Asteraceae (Coutinho et al. 2021). Furthermore, browning has also been observed in marigold before (Kaňuková et al. 2024). The browning did not impede our work as long as calli and cultures were subcultured in a timely manner.

As it was planned to use CR1 for the production of recombinant proteins, exporting the proteins to the cell culture medium would facilitate the purification process. Therefore, the proteolytic activity of the SN was of interest. Zymography using gelatin as a target showed very little proteolytic degradation, which was distinctly different from the SN of BY2 cell culture of the same age and cell density that was analyzed in the same experiment for comparison. When BY2 cells are used for the production of recombinant proteins, the proteases in the culture supernatant have been a challenge (Schiermeyer et al. 2005). Thus, the low amount of gelatin degradation by the CR1 SN is an advantageous feature of the newly established plant suspension culture. We did not analyze the degradation of brazzein produced in CR1 and exported to the culture supernatant; however, because brazzein is a small and tightly packed protein containing four intramolecular disulfide bonds, it is highly resistant to proteolytic degradation (Kohmura et al. 1996) and would thus have been an unsuitable target for degradation studies.

CR1 was transformed using a construct with two independent expression cassettes: one for DsRed targeted to the ER and the other for brazzein targeted to the apoplast. This choice combined the easy visual detection of transformation events with high expression and existing data from other plant production systems for the fluorescent DsRed, along with the challenges (small molecule, no commercial antibody for detection available) and industrial potential of the sweet protein brazzein (Lamphear et al. 2005). We did not use codon optimization on the two genes as there is only weak data available on *Calendula officinalis* codon usage (https://www.kazusa.or.jp/codon/cgi-bin/showcodon.cgi?species=41496) and analysis of codon usage in the closely related *Helianthus annus* (https://www.kazusa.or.jp/codon/cgi-bin/showcodon.cgi?species=4232) showed no truly “rare” codons. Knowing that changes in codon usage can have both negative and positive effects (Rozov & Deineko 2019), we decided to use the genes with their original codons.

Tetrameric DsRed, the same as used here, has been produced in BY2 plant cell packs (transient transformation), where approximately 40 mg/kg cell biomass was achieved with ER targeting (Opdensteinen & Buyel 2022). However, markedly more—around 200 mg/kg cell biomass—was produced in the same system when DsRed was targeted to the cytosol. The best result for DsRed production in CR1 was 1322 mg/kg cell (transformation event 155), approximately seven times higher than in the best BY2 cell scenario. Although transformation event 155 was a slowly growing culture and thus slightly impractical, the average production in the seven analyzed cultures (Table 1) was 533 mg/kg cell biomass, which is still more than twice as much as in the transiently transformed BY2 cells. The cells analyzed here were stably transformed; therefore, the comparison is slightly skewed. However, it is generally assumed that recombinant protein production is higher in transiently transformed plant cells than in stably transformed ones (Xu & Zhang 2014), which likely underestimates the capacity of the CR1 cells.

It was noticed that the transformation events that produced a lot of DsRed displayed lower growth rates (transformation event 155 being the most prominent example). This is an interesting result that will need more detailed analysis; however, in the context of this dataset, we did not look into the causal relationship between the two parameters.

DsRed quantification was performed in both the cells and SNs of CR1 transformation events. The small amount of DsRed detected in the SNs indicates that most of the cultures consisted of intact cells at harvest.

We observed that the different transformation events varied substantially in biomass accumulation. For the measurement of DsRed in the cells, this was not of importance; however, for the quantification of brazzein in the SNs, the varied biomass accumulation seemed to confuse the growth and productivity of the transformation events. For that reason, some cultures were cultivated longer than the usual 7 days before harvest. We noted that after 15 days, even cultures with low cell density started to brown, and therefore this was the margin used here.

The selected transformation events that were analyzed for DsRed production were also analyzed for brazzein production. To this end, we prepared a brazzein standard. This brazzein standard was generated from SN of transformation event 74 by cation exchange chromatography with subsequent ultrafiltration, following a previously described procedure (Lamphear et al. 2005). The resulting highly enriched brazzein fraction still contained minor impurities; however, complete purity was not essential for its use as reference material. The brazzein standard was analyzed for integrity by top-down LC–MS/MS, leading to the same molecular weight of 6473 Da as found in *Pentadiplandra brazzeana* B (Ming and Hellekant 1994) and was quantified by a bottom-up LC–MS/MS analysis using a commercial stable isotope-labeled reference peptide of brazzein. SDS-PAGE of the SNs showed focused protein bands at 6.5 kDa in all analyzed transformation events but not in the Wt. To obtain such clear signals for brazzein, it was necessary to concentrate the SNs, perform protein alkylation and use a Schägger gel. Once established, this procedure is easily applied and can be used for a quick pre-screen of many transformation events to preselect suitable candidates for further analysis or quantification. Given the relatively small size of brazzein (6.5 kDa) and the simplicity of the sample matrix in SNs, we adopted a top-down strategy for the quantification of brazzein in the SNs of the selected transformation events. This approach minimizes errors introduced by extensive sample preparation and avoids erroneous quantification due to incomplete digestion or modified peptide species, since only intact protein isoforms are measured (Donnelly et al. 2019). Such a strategy has been used before (Olsen et al. 2016) for a different small protein (with 36 amino acids), and the limitation of not being able to use an isotopically labeled internal standard could be countered by utilizing the brazzein standard generated here and an external calibration. This approach has the benefit of detecting only full-size molecules, with the limitation that it does not detect modified variants of brazzein. Since a preliminary untargeted, data-dependent acquisition of each sample was performed without detecting any significant amounts of brazzein-related variants, the method used here is justified.

Not many publications exist where the production of brazzein has been quantified in plant suspension cells; however, brazzein was produced in carrot cells (Han et al. 2022), where after 12 days, 1.2 µg/g fw of brazzein was recorded. In this study, we produced 259 µg/g fw after 15 days, which is approximately 200 times more. Furthermore, the brazzein produced here is without a tag and in the SN, while Han et al. (2022) produced the sweet protein in the cells containing a flag-tag. Attachment to the N-terminus or C-terminus of brazzein can change the taste and can completely obliterate the sweetness of the molecule (Jafarian et al. 2020). For potential industrial use of brazzein, the primary production host is yeast, where 90 mg/L (Poirier et al. 2012) and more than 300 mg/L (Neiers et al. 2021) have been reported, both values clearly exceeding the 24.5 mg/L produced here. We assume that the production of brazzein in transformation event 74 could be improved through process optimizations; anyhow, the purpose of this work was not the production of brazzein but rather showcasing the potential of CR1.

We checked for consistency of TSP values measured (Table S1) and noted that the values of Wt, event 74 and event 155 correlated similarly with the amount of cell biomass in the cultures (i.e., 4.4–5.2 mg TSP/g cell fw), while the culture of event 16 was distinctly deviating from this value (2.5 mg TSP/g cell fw). It is assumed that this is due to the late growth stage of culture 16, where no longer cell division but water uptake is responsible for increase in cell biomass (Godoy-Hernández & Vázquez-Flota 2006).

It might seem strange that some of the analyzed transformation events produced a large amount of DsRed but only a small amount of brazzein. In fact, the molar total production ratio of DsRed to brazzein varied between 1:2 (event 74) and 1:0.24 (event 16), so the idea that the amount of DsRed produced is an indicator of the production amount of the second protein from the tandem construct does not hold true. Similar scenarios (Peach & Velten 1991) have shown the same outcome. Furthermore, multiple insertions and/or partial insertions of the used T-DNA might be responsible for this result. Multiple and partial insertions of the T-DNA are common occurrences (Kirchhoff et al. 2020) and can lead to asymmetric expression when the construct used for transformation codes for two different proteins. We can use DsRed as an indicator for successful transformation, but the magnitude of DsRed production is not correlated to the production of the second protein that the tandem construct codes for.

We did not analyze transgene stability per se; however, 33 months after the transformation, all seven analyzed calli and 41 calli that were not analyzed are still alive and expressing DsRed.

With the data provided, CR1 has been established as a rapidly growing cell culture with minor protease activity in the cell culture supernatant. This plant suspension culture has very good recombinant protein production capacities, as shown for DsRed in the ER (up to 1.3 mg/g fw) and also for brazzein in the culture supernatant (up to 24.5 µg/mL), and will be useful in future plant biotechnology setups.

## Supplementary Information

Below is the link to the electronic supplementary material.

Supplementary file1 (DOCX 18 KB)

Supplementary file2 (PDF 389 KB)

## Data Availability

All data in this study are available from the corresponding author on reasonable request.
