# Supplementary material for: Marigold suspension cell culture as production system for recombinant DsRed and brazzein
Source: Plant Cell Rep. 2026 Sep 30;45(10):317. doi: 10.1007/s00299-026-03982-8 (PMC13627208; doi:10.1007/s00299-026-03982-8)
Supplement: Supplementary file 1 [file 299_2026_3982_MOESM2_ESM.pdf]

**Plant cell reports:** Marigold suspension cell culture as production system for recombinant DsRed and Brazzein

Matthias Buntru, Alexander Croon, Jürgen Müller, Marika Hanke and Helga Schinkel*

*Corresponding author: Helga Schinkel, helga.schinkel@ime.fraunhofer.de

Fraunhofer Institute for Molecular Biology and Applied Ecology IME, Forckenbeckstrasse 6, 52074 Aachen, Germany

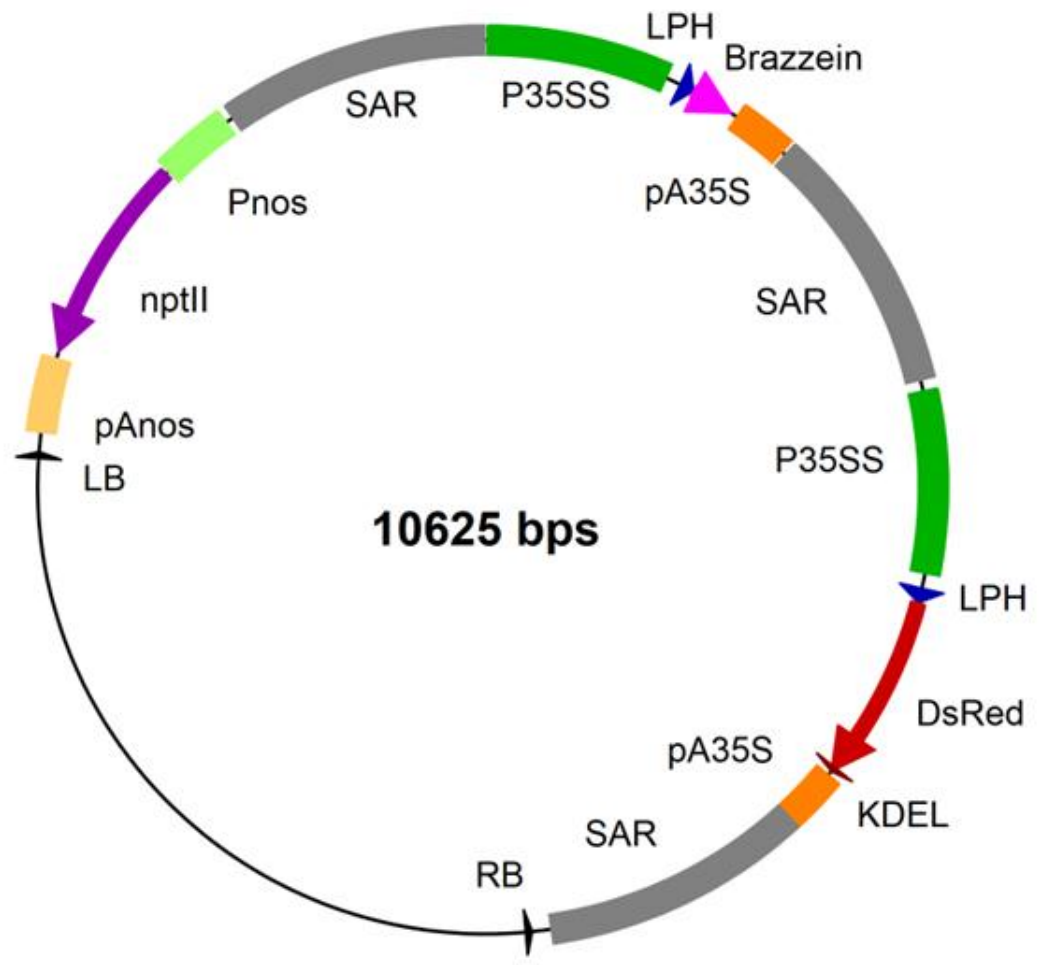


**Fig S1** Vector map of pTRAkc_LPH-Braz-rfp-ERH. The vector pTRAkc_LPH-Braz-rfp-ERH was used for transformation of suspension cells CR1. It contains two separate expression cassettes for brazzein and DsRed, both regulated by an enhanced 35s promoter and terminated by a 35s terminator. Both proteins have the LPH signal peptide for transport into the apoplast at their 5' end, DsRed has also a KDEL-sequence at the 3'end for retention in the endoplasmastic reticulum. Besides the two described cassettes a third cassette coding for neomycin phosphotransferase II, i.e. confering kanamycin resistance of transformed cells, under control of the nopaline synthase promoter and terminated by the nopaline synthase terminator, is present in the vector. All three cassettes are separated by scaffold attachment regions. RB: right border; SAR: scaffold attachment regions; pA35S: 35s terminator; KDEL: Endoplasmic reticulum retention sequence; DsRed: gene coding for red fluorescent protein from *Discosoma* spec; LPH: Signal peptide for export to the apoplast (Schiermeyer et al, 2005); P35SS: enhanced 35s promoter; Brazzein: gene coding for sweet protein brazzein; Pnos: Nopaline synthase promoter; nptII: gene coding for neomycin phosphotransferaseII; pAnos: Nopalin synthase terminator; LB: left border

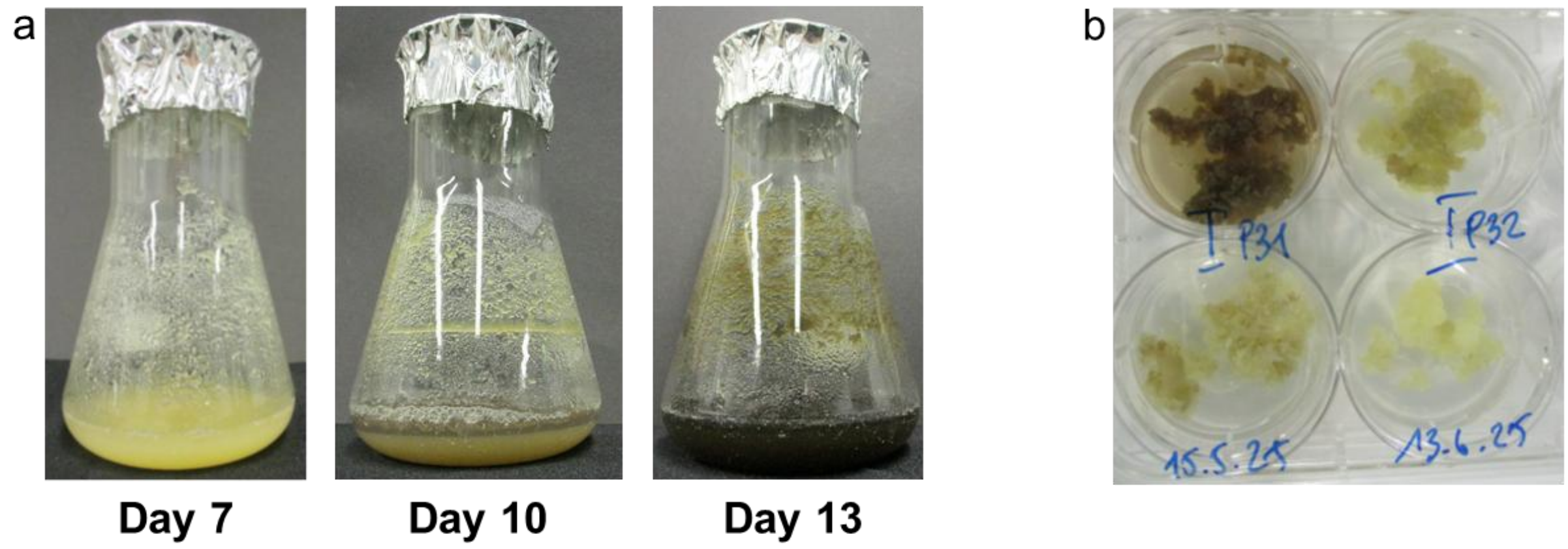


**Fig S2** Browning of CR1 culture and calli. CR1 culture passaged once weekly (day 7) is quickly turning brownish-black when not passaged. **a** The same culture (passage 126) is yellowish on day 7 (when passage happens), but brownish three days later (day 10) and nearly black another three days later (day 13). **b** Calli were passaged once a month, the picture was taken 25 days after the last passage

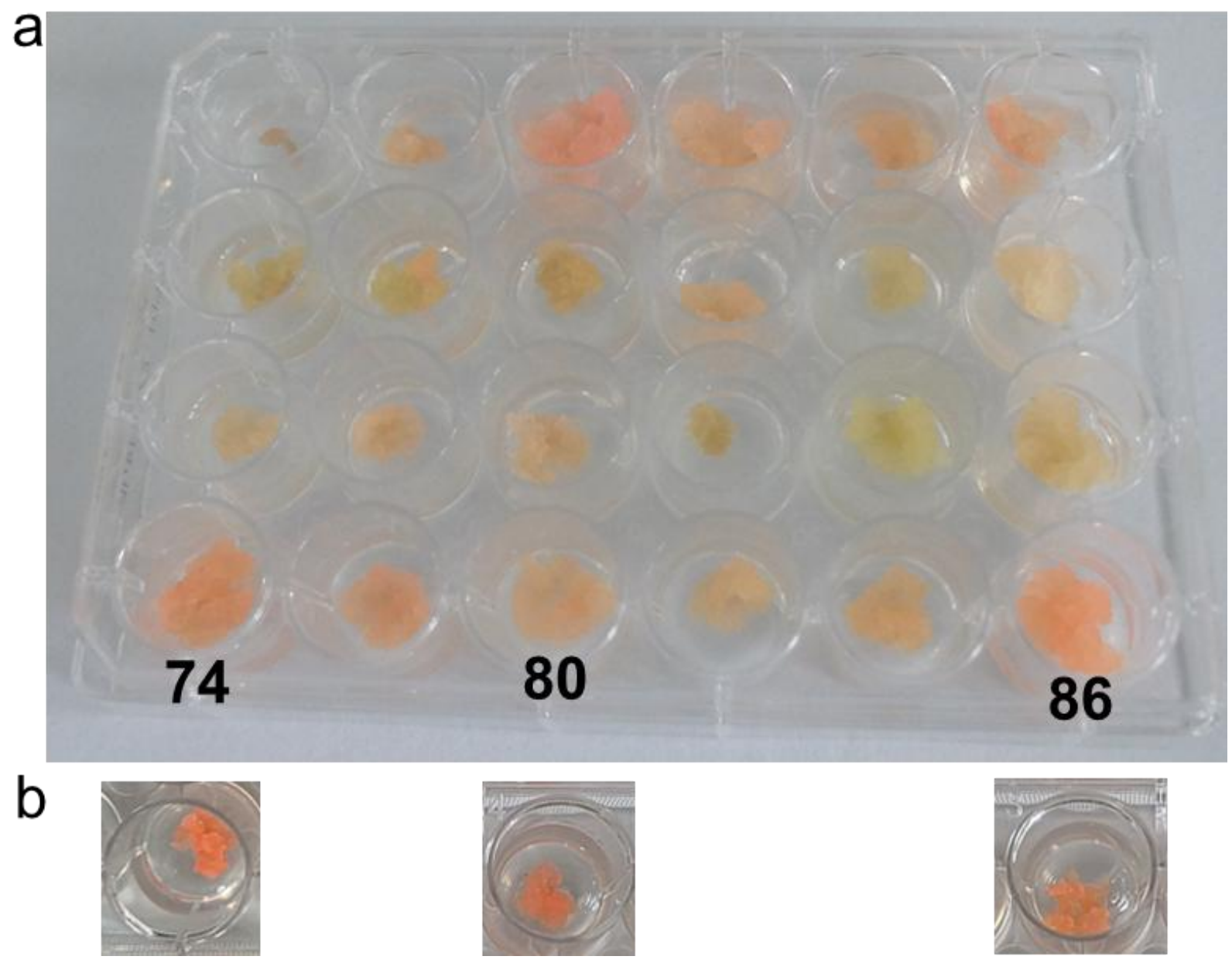


**Fig S3** Transformation events show variation in growth and DsRed expression. The picture was taken 17 days after passaging (passage 11), the three numbered calli are of events that were analysed for both DsRed and brazzein accumulation. (b) two years later the same three calli were photographed 8 days after passage 49.

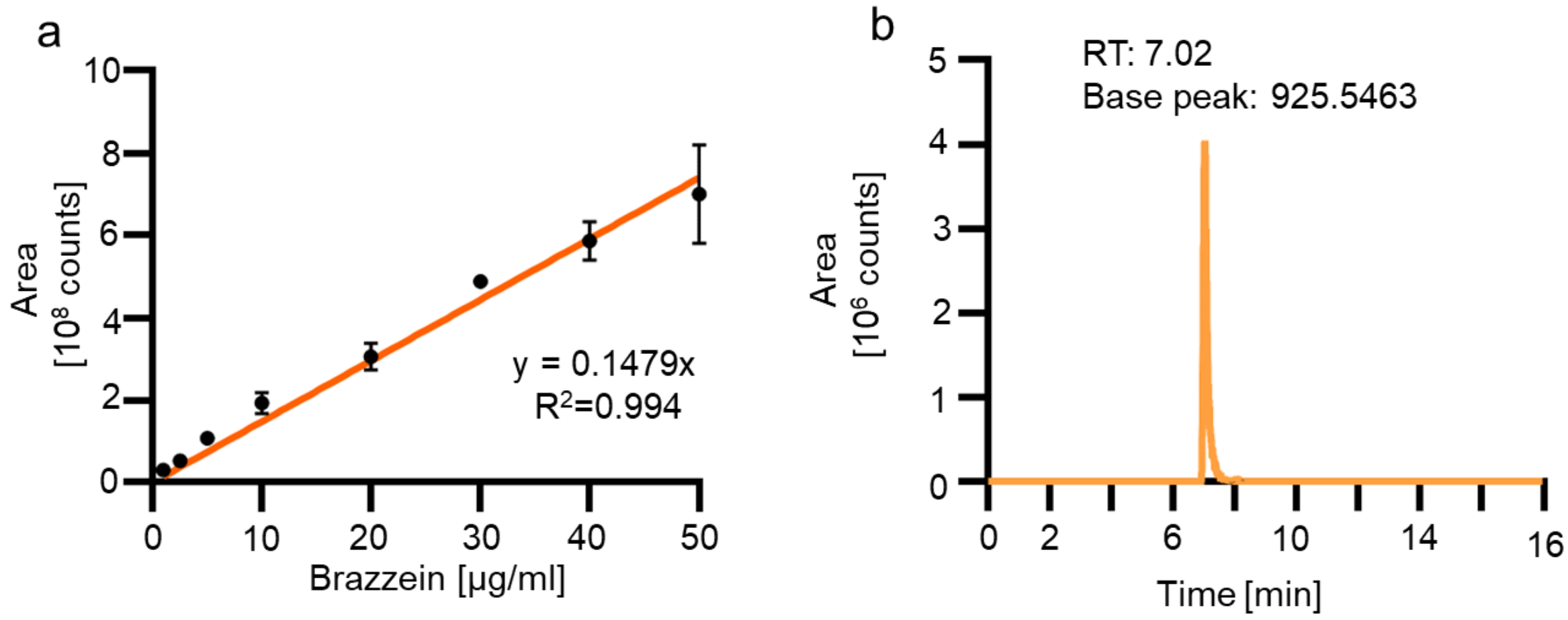


**Fig S4:** Exemplary top-down quantification and linear regression analysis of brazzein in supernatant 80 by LC-MS. **a** Calibration of LC-MS by linear regression from 1.0 to 50 µg of brazzein/mL (10 to 500 ng on column) spiked in Wt *C. officinalis* supernatant. **b** TIC chromatogram of targeted selected ion monitoring of SN 80 showing the peak area of brazzein at 7.02 min with a base peak at *m/z* 925.5463

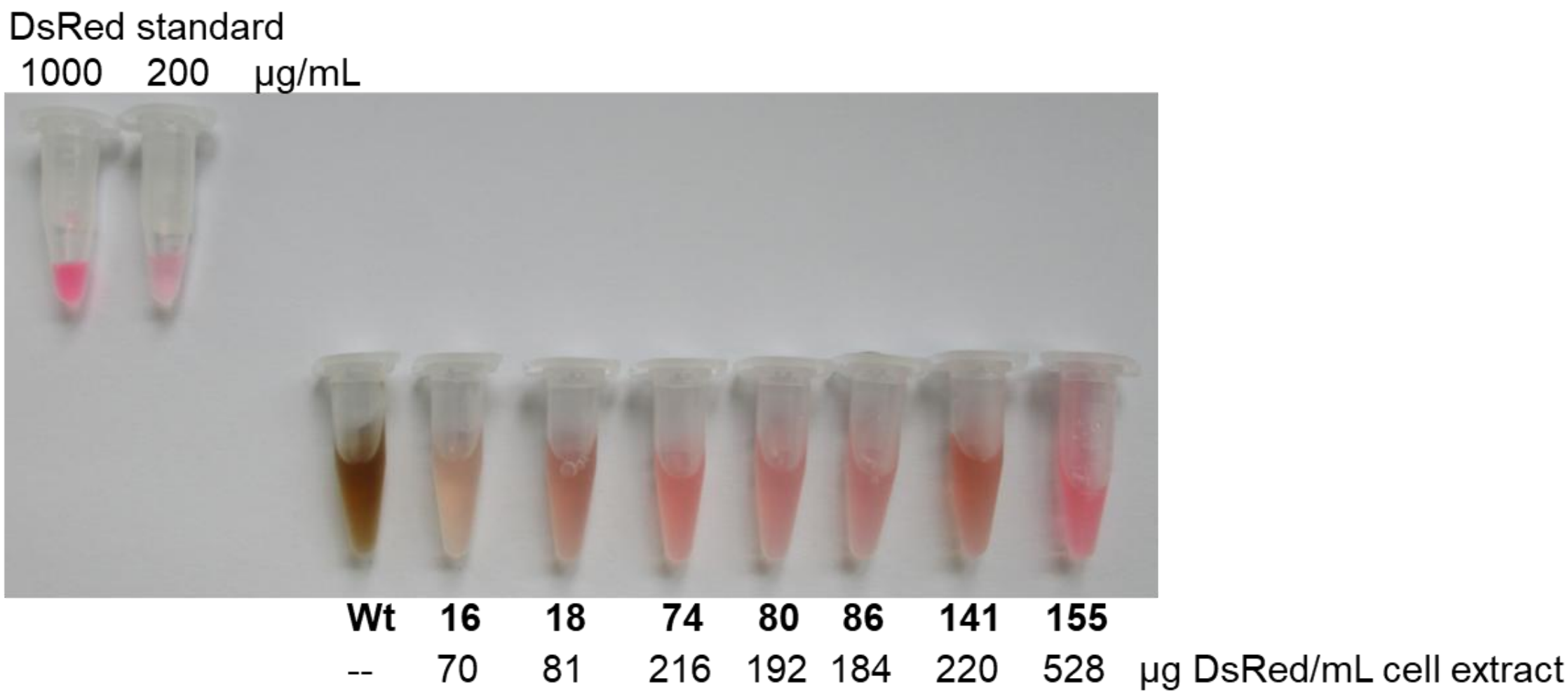


**Fig S5:** Cell extracts show the high production of DsRed in transformation events. The picture was taken of one (out of four) cell extracts that were quantified spectrophotometrically by comparison to a DsRed standard. Two tubes with the DsRed standard at 1000 µg/mL and 200 µg/mL are in the upper left corner, while the extracts that were measured are below. The number of the transformation event is given directly under the picture and the measured DsRed concentration (µg/mL) in that particular extract is provided in the line below

Table S1: Total soluble protein (TSP), total recombinant protein produced, and cell biomass in transformation events 16, 74 and 155 and in the wild type (Wt). Each culture had a volume of 50 ml.

| Transformation event | TSP [mg] | Brazzein [mg] | DsRed [mg] | Cells [g fw] |
|---|---|---|---|---|
| Wt | 36 | --- | --- | 7.0 |
| 16 | 34 | 0.14 | 2.4 | 13.7 |
| 74 | 19 | 0.93 | 2.0 | 3.6 |
| 155 | 16 | 0.45 | 5.0 | 3.6 |
