# Supplementary material for: Marigold suspension cell culture as production system for recombinant DsRed and brazzein
Source: Plant Cell Rep. 2026 Sep 30;45(10):317. doi: 10.1007/s00299-026-03982-8 (PMC13627208; doi:10.1007/s00299-026-03982-8)
Supplement: Supplementary file 2 [file 299_2026_3982_MOESM1_ESM.docx]

Supplemental text

**Material and methods**

S1: Preparation and Validation of a Brazzein Standard

The SN of a 200 mL culture from transformation event 74 was harvested after 15 days of cultivation via vacuumfiltration. Dialysis of 70 mL of SN against citrate buffer (5 mM, pH 4) was performed overnight at 4°C, followed by filtration (0.22 µm) and pH -adjustment to 4.0 using citric acid. Cation exchange chromatography was conducted using a 1 mL HiTrap SP FF column (Cytiva, Freiburg, Germany) coupled to an Äkta Explorer 100 chromatography system, with 12 mM trisodium citrate (buffer A) and 12 mM trisodium citrate with 1 M NaCl (buffer B). Elution fractions of 1 mL were collected and analyzed by SDS-PAGE for the presence and purity of brazzein. The five fractions with the strongest brazzein signal were combined and dialyzed against phosphate-buffered saline (PBS). The sample was centrifuged in a filter device (Vivaspin Turbo 15, 10,000 MWCO, Sartorius, Stonehouse, UK). The filtrate, containing brazzein, was retained, aliquoted, and frozen at -80°C.

Integrity testing of the brazzein entity in the standard was performed using a top-down liquid chromatography-tandem mass spectrometry (LC-MS/MS) approach to determine the molecular weight of the principal peak. For this analysis, 100 µL of the standard was precipitated with cold acetone, and the protein pellet was reconstituted in 100 µL of 8 M urea and 50 mM ammonium bicarbonate (denaturing buffer). It was then desalted using 100 µL C18 tips (87784, Thermo Fisher Scientific, Waltham, MA, USA) and concentrated by SpeedVac. The final volume was adjusted to 50 µL using 5% (v/v) acetonitrile with 0.1% (v/v) formic acid (FA). LC-MS/MS was conducted on an Ultimate 3000 UHPLC coupled to a Q Exactive HF Orbitrap mass spectrometer (Thermo Fisher Scientific). Liquid chromatography (LC) was performed using a C18 column (2.1 x 100 mm, 80 Å; Accucore aQ) (Thermo Fisher Scientific) at 30°C and 0.1% (v/v) FA (eluent A) and 0.1% (v/v) FA in acetonitrile (eluent B) at a flow rate of 0.3 mL/min. A 10 µL sample was injected. Separation began at 0% (v/v) of eluent B for 1 minute, followed by a linear gradient from 0 to 50% (v/v) of eluent B over 11 minutes. The mass spectrometer (MS) was operated in positive ionization mode using a heated electrospray ionization source. Brazzein was monitored in data-dependent acquisition mode with a scan range of *m/z* 300 to 1500. The resolution was 60,000 at *m/z* 200 for full MS. The isolation window was *m/z* 2.0, and the normalized collision energy (NCE) was set to 30. The resolution of tandem MS was 15,000 at *m/z* 200. Data processing and peak deconvolution were performed using the Xtract deconvolution algorithm of the Freestyle Software (ver. 1.8.65.0; Thermo Fisher Scientific).

A bottom-up approach was used to determine the amount of brazzein in the standard. For this, 100 µL of the standard was precipitated and dried as described above, and then reconstituted in 10 µL of denaturing buffer. Two microliters of a stable isotope-labeled peptide CQLANQCNYDCK* (JPT Peptide Technologies GmbH, Berlin, Germany) were added to the standard before reduction by adding 4 µL of 80 mM dithiothreitol (DTT), followed by incubation at 60°C for 60 minutes. Alkylation of the proteins was achieved by adding 4 µL of 200 mM iodoacetamide to the sample (30 minutes at room temperature) and quenching with 4 µL of 60 mM DTT. For enzymatic digestion, the sample was diluted by adding 66 µL of ammonium bicarbonate buffer (50mM, pH 8.0) and 2 µL of trypsin/LysC (250 ng/µL). The digestion was carried out overnight at 37°C and stopped by the addition of 8 µL of 10% (v/v) trifluoroacetic acid. Peptides were desalted and dried as described above. The final volume was adjusted to 50 µL using 0.1% (v/v) FA. For LC-MS/MS analysis samples were additionally diluted 1:10 using 0.1% (v/v) FA.

LC was conducted as described above for the integrity of the brazzein standard, except the elution gradient ranged from 0-50% in 20 minutes. Peptides were detected using parallel reaction monitoring in positive ionization mode with a resolution of 30,000 at *m/z* 200. The NCE was set to 27, the isolation window was *m/z* 1.2, and the automatic gain control target was 2x10^5^. The naturally occurring peptide CQLANQCNYDCK was monitored at *m/z* 787.3096, while the isotopically labelled peptide was monitored at *m/z* 791.3096. Data processing and quantification were achieved by calculating the light-to-heavy ratio of the peptide CQLANQCNYDCK using the Skyline Software (ver. 24.1.0.414; MacLean et al., 2010).

MacLean B, Tomazela DM, Shulman N, Chambers M, Finney GL, Frewen B, Kern R, Tabb DL, Liebler DC, MacCoss, MJ. (2010). Skyline: an open source document editor for creating and analyzing targeted proteomics experiments. Bioinformatics 26(7), 966–968. <https://doi.org/10.1093/bioinformatics/btq054>
